# Neural mechanisms of acupuncture for peripheral facial nerve palsy: A protocol for systematic review and meta analysis

**DOI:** 10.1097/MD.0000000000033642

**Published:** 2023-05-05

**Authors:** Changwoo Seon, Dong Hyuk Lee, Bo-In Kwon, Jun-Sang Yu, Sang Kyun Park, Yeonju Woo, Joo-Hee Kim

**Affiliations:** a Department of Acupuncture and Moxibustion Medicine, College of Korean Medicine, Sangji University, Wonju-si, Republic of Korea; b Department of Anatomy, College of Korean Medicine, Sangji University, Wonju-si, Republic of Korea; c Research Institute of Korean Medicine, Sangji University, Wonju-si, Republic of Korea; d Department of Pathology, College of Korean Medicine, Sangji University, Wonju-si, Republic of Korea; e Department of Sasang Constitutional Medicine, College of Korean Medicine, Sangji University, Wonju-si, Republic of Korea; f Department of Meridian and Acupoints, College of Korean Medicine, Sangji University, Wonju-si, Republic of Korea; g Department of Physiology, College of Korean Medicine, Sangji University, Wonju-si, Republic of Korea.

**Keywords:** acupuncture, Bell’s palsy, functional magnetic resonance imaging, neuroimaging, peripheral facial nerve palsy, systematic review

## Abstract

Peripheral facial nerve palsy (PFNP) is a cranial neuropathy that occurs when the seventh facial nerve is damaged. PFNP seriously affects patients’ quality of life, and approximately 30% of patients suffer from sequelae, such as unrecovered palsy, synkinesis, facial muscle contracture, and facial spasm. Many studies have confirmed the effectiveness of acupuncture for the treatment of PFNP. However, the specific mechanism remains unclear and needs to be further explored. Therefore, the purpose of this systematic review is to investigate the neural mechanisms underlying acupuncture treatment for PFNP using neuroimaging methods.

**Methods:**

We will search all published studies from inception to March 2023 using the following databases: MEDLINE, Cochrane Library, EMBASE, CNKI, KMBASE, KISS, ScienceON, and OASIS. All clinical studies evaluating the effectiveness of acupuncture for treating PFNP using functional neuroimaging will be selected without language restrictions. Two reviewers will independently conduct the study selection, data extraction, and risk of bias assessment, according to a predetermined protocol. The outcomes, including the types of functional neuroimaging techniques, brain function alterations, and clinical outcomes, such as the House-Brackmann scale and Sunnybrook Facial Grading System, will also be analyzed. Coordinate-based meta-analysis and subgroup analyses will be performed if possible.

**Results:**

This study will analyze the effect of acupuncture on brain activity alterations and clinical improvement in patients with PFNP using functional neuroimaging.

**Conclusion:**

This study will provide a comprehensive summary and help elucidate the neural mechanisms of acupuncture treatment for PFNP.

**PROSPERO registration number:**

CRD42022321827.

## 1. Introduction

Peripheral facial nerve palsy (PFNP) is the most common cranial neuropathy and can occur when the 7th cranial nerve, including its motor nucleus, is damaged for various reasons.^[1]^ Although there are a few known causes for PFNP, most cases are idiopathic. Bell’s palsy (BP) is an idiopathic condition in which the 7th cranial nerve is affected and causes unilateral facial muscle weakness or paralysis that is acute in onset.^[2]^ Since the exact cause of BP is still unclear it is usually diagnosed only when other potential etiologies of facial paresis, such as infection, trauma, neoplasms, and congenital or syndromic problems, are clinically ruled out.^[3]^

The incidence of BP is estimated at 20 to 25 cases per 100,000 population per year.^[4]^ Country based epidemiological studies have reported an annual incidence of more than 60,000 cases of BP in the United States and about 420,000 in China.^[5]^ The clinical features of BP include sudden onset, unilateral weakness of the facial muscles that may be accompanied by pain behind the ear, taste disorders, smell disorders, and hyperacusis depending on the location of the lesion along the course of the facial nerve.^[6]^

Many patients with BP generally recover facial muscle function. However, some patients have an incomplete recovery, and sequelae such as synkinesis, contracture, spasm, and crocodile tear syndrome may remain.^[7]^

The facial nerve is mainly a motor nerve that emerges from the facial nerve nucleus of the pons. Its travel path is longer and narrower than that of other cranial nerves, so it can be easily damaged by conditions such as otitis media, trauma, and infection. Some studies have shown that the density of capillaries in the endoneurium corresponds to the vulnerability to ischemic nerve injury, suggesting the possibility of peripheral nerve vulnerability due to the density of the endoneurial capillaries.^[8]^ Potential mechanisms associated with nerve damage in idiopathic facial nerve palsy include ischemia and demyelination induced by inflammatory changes.^[9]^ However, despite several previous reports to explore underlying mechanisms causing facial nerve palsy, the evidence is not sufficient.

Acupuncture has been used to treat various neurological disorders, including facial palsy, and many previous studies have reported its clinical effectiveness and safety. The brain has the ability to enhance adaptive changes and cerebral reorganization that are important for functional recovery.^[10]^ Functional neuroimaging methods including functional magnetic resonance imaging (fMRI), positron emission tomography (PET), and single-photon emission computed tomography (SPECT) can be widely applied not only to detect brain alterations in various diseases, but also to identify the effects of interventions, such as acupuncture. Among them, fMRI is the most widely used modality in research because of its excellent spatiotemporal resolution and noninvasive nature.^[11,12]^ Using fMRI, some changes in cortical activation have been observed in patients with varied degrees of acute PFNP.^[10]^ Previous fMRI studies have also shown cortical reorganization of this network in both central and peripheral palsy.

To date, several clinical studies using functional neuroimaging have been conducted to reveal the neural mechanisms underlying acupuncture treatment in PFNP. However, the results have been inconsistent due to the heterogeneity of the methodology, quality, and design of the studies. In order to glean a better understanding of the effects of acupuncture treatment on PFNP based on functional neuroimaging, a review of the existing literature was planned. Therefore, this systematic review aims to investigate the neural mechanisms of acupuncture treatment in PFNP using neuroimaging methods. In this study, clinical trials that have utilized acupuncture as a treatment modality for PFNP and employed functional neuroimaging will be systematically evaluated to elucidate the neural mechanisms induced by acupuncture treatment for PFNP.

## 2. Methods

### 2.1. Study design and registration

The protocol for this systematic review has been registered on the International Platform of Registered Systematic Review and Meta-analysis Protocols (PROSPERO, registration number: CRD42022321827). All procedures for the study will be conducted and reported in accordance with the Preferred Reporting Items for Systematic Reviews and Meta-Analysis (PRISMA) Protocol statement guidelines.^[13]^

### 2.2. Eligibility criteria

#### 2.2.1. Types of participants.

This review will include studies on patients diagnosed with PFNP, regardless of the stage. No constraints will be placed on age, gender, race, and/or economic status.

#### 2.2.2. Types of interventions.

The intervention methods include acupuncture without any limitations on the acupuncture type, techniques, stimulation method, etc.

#### 2.2.3. Types of outcomes.

The types of functional neuroimaging techniques and neuroimaging results of indices such as functional connectivity, regional homogeneity (ReHo), and fractional amplitude of low-frequency fluctuation (fALFF) in fMRI will be included. Clinical outcomes, such as the House-Brackmann (HB) scale and Sunnybrook Facial Grading System, will also be included and analyzed in this review.

#### 2.2.4. Types of studies.

The types of studies will be all clinical studies including randomized controlled trials, case series, and observational studies investigating the effect of acupuncture on brain changes induced by peripheral facial nerve palsy using functional neuroimaging. Experimental studies using animal models, letters, editorials, and/or conference proceedings will be excluded.

### 2.3. Search strategies

A systematic search of electronic databases including PubMed, Cochrane Library, EMBASE, CNKI, Korean Medical database, Korean Studies Information Service System, ScienceON, and Oriental Medicine Advanced Searching Integrated System will be performed using search terms as follows: “peripheral facial nerve palsy,” “Bell’s palsy,” “acupuncture,” “acupoint,” “acupuncture therapy,” “neuroimaging,” “fMRI,” “functional MRI,” “MRI,” “PET,” “SPECT,” “functional connectivity,” “blood oxygen level dependent,” and so on. The specific search strategy for PubMed is presented in Table 1, and the search strategy for other databases will be adjusted for each database. All studies published until March 2023 will be searched without language restrictions.

**Table 1:** The search strategy for PubMed.

Number	Search terms
1	Facial nerve palsy
2	Bell’s palsy
3	Peripheral facial nerve palsy
4	Facial palsy
5	Idiopathic facial palsy
6	OR #1-5
7	Acupuncture
8	Acupuncture therapy
9	Acupoints*
10	Electroacupuncture
11	auriculopuncture
12	OR #7-11
13	Neuroimag*
14	Magnetic resonance imag*
15	MRI
16	PET
17	DTI
18	fMRI
19	Resting state
20	Functional imag*
21	Functional connectivity
22	SPECT
23	Blood oxygen level dependent
24	BOLD
25	Regional homogeneity
26	ReHo
27	Amplitude of low-frequency fluctuation
28	ALFF
29	fALFF
30	Voxel-based analys*
31	Centrality
32	ICA
33	Independent component analysis
34	ROI
35	Region of interest
36	OR #13-35
37	#6 AND #12 AND #36

### 2.4. Data collection and analysis

#### 2.4.1. Selection of studies.

**Figure 1.**
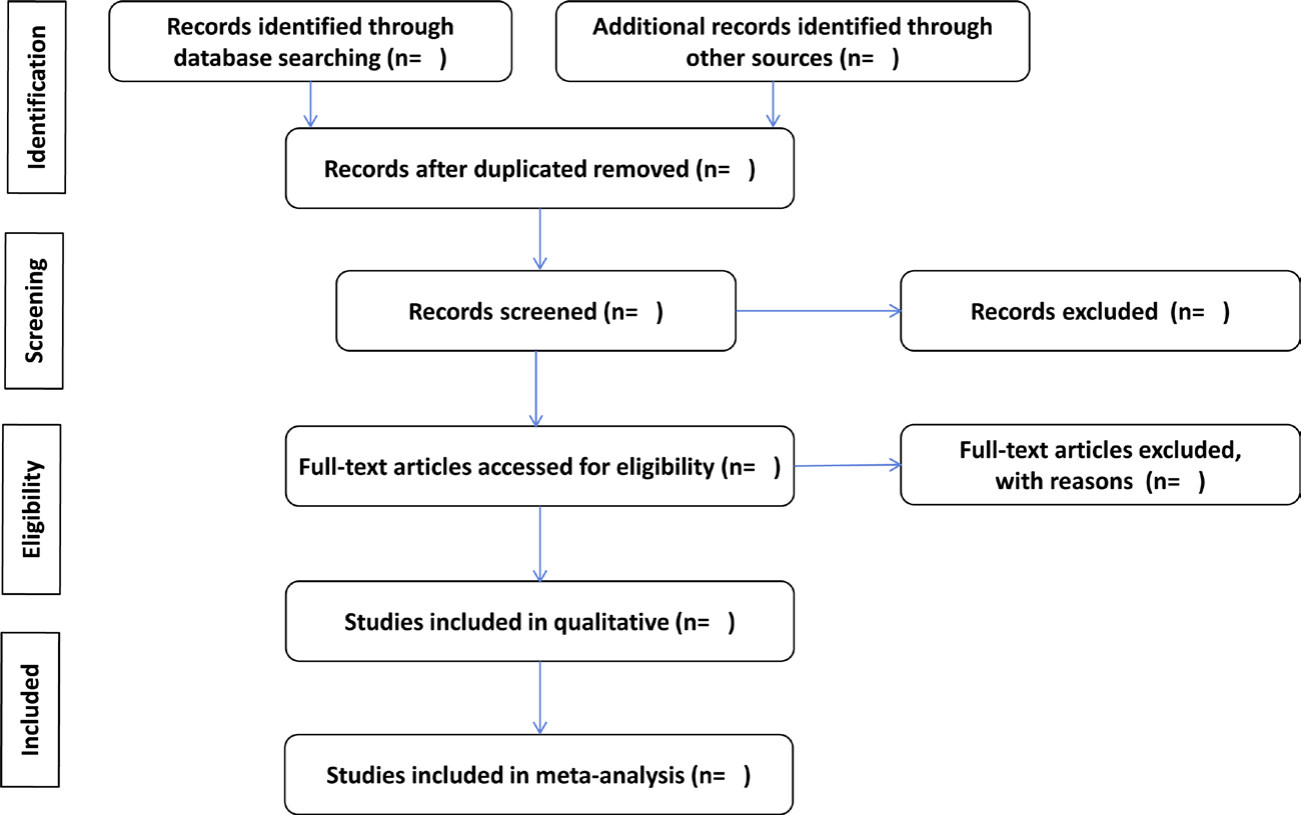
Flow chart of the study selection.

Two reviewers (C.W.S. and D.H.L.) will independently screen and select pertinent studies from the search results. After merging the search results from the databases using EndNote 20, duplicate records will be removed. The reviewers will exclude obviously irrelevant studies by screening titles and abstracts, examining the full text of potentially relevant articles, and making final decisions regarding study inclusion using predefined criteria. The reasons for exclusion will be documented. Any disagreement between the two reviewers will be resolved through discussions. If the 2 reviewers fail to reach a consensus, a third reviewer (J.H.K.) will make the final decision. Figure 1 shows the study selection flow.

#### 2.4.2. Data extraction.

Two reviewers will screen the study, extract the data independently, and discuss the discrepancies to reach an agreement.

The pre-determined data extraction form will include the following information: identification information (title, name of the first author, year of publication, etc.), general information (country, sample size, study design, etc.), participant characteristics (age, sex, race, BP stage, etc.), treatment and control group intervention methods (methods of acupuncture treatment, length of needle, de-qi, treatment period, etc.), and clinical and neuroimaging outcomes.

#### 2.4.3. Assessment of risk of bias.

The risk of bias of each included randomized controlled trials will be independently evaluated by 2 reviewers (C.W.S. and D.H.L.) using the risk of bias assessment method of the Cochrane system reviewer’s handbook, which focuses on selection bias (random sequence generation and allocation concealment), implementation bias (blinding of participants and personnel), evaluating bias (blinding of outcome assessment), attrition bias (incomplete outcome data), reporting bias (selective study outcomes reporting), and other biases.

#### 2.4.4. Data synthesis and statistical analysis.

Coordinate-based meta-analysis and GingerALE software version 3.0.2 (http://brainmap.org/ale/) will be utilized to carry out the analyses. The convergence of brain areas will be analyzed by testing against the null-hypothesis of random spatial maps. Regions of interest (ROIs) with significant alterations of brain activation in the PFNP, significant changes in activation induced by acupuncture treatment in the included studies, and neural correlates of clinical measurements or improvement will be summarized. The peak coordinates in the Talairach space will be transformed into the MNI space (or vice versa). The standard thresholds will be set as uncorrected *P* < .005 and voxel size ≥ 10 to balance the sensitivity and specificity. For missing data, we will try to contact corresponding authors for further original data, and conduct sensitivity analysis to address potential effect of missing data if necessary. When appropriate studies are included, subgroup analysis will be performed to assess the potential causes of heterogeneity.

## 3. Discussion

BP is the most common form, accounting for approximately 50% to 60% of all PFNP.^[10]^ The cause of BP is unknown, but typically viral infection that means reactivation of the herpes virus in the geniculate ganglion of the facial nerve, vascular ischemia, or autoimmune disease are presumed to be its pathogenesis.^[14,15]^ Symptoms and signs appear from the fact that the facial nerve is a mixed nerve that is a mixture of motor fibers that control the expression muscles of the face including the stapedius muscle and sensory fibers that are involved in taste, and that it contains parasympathetic nerve fibers that are distributed in the submandibular, sublingual and lacrimal glands.^[16]^ In addition, patients are under extreme stress due to the sequelae. In 29% of patients with BP, residual paresis symptoms appear as sequelae, contracture in 17%, and synkinesis in 16%.^[17]^ Some patients with serious sequelae show psychosocial problems such as difficulty with eating, drinking, and talking and fear of human relationships.^[18]^

Acupuncture is recognized as an effective and safe treatment for a range of disorders, including PFNP.^[19,20]^ Studies on changes caused by acupuncture stimulation in brain functional connectivity in BP patients are currently lacking.^[21]^ Neuroscience can be studied using neuroimaging techniques that help non-invasively examine the human brain. Recently, with the rapid development of imaging technology, various brain imaging technologies, such as Positron Emission Tomography, Electroencephalogram and Transcranial Magnetic Stimulation, have been developed. Functional Magnetic Resonance Imaging is also performed. Among them, functional neuroimaging is widely used in relation to the mechanism of acupuncture treatment.^[22]^ Zhao et al^[23]^ reported that acupuncture may control the functional connection of the functional brain network and functional activities of the cognitive brain area. BP is a peripheral nerve injury that affects the motor nerves on one side of the face. One study showed that BP induced different changes in Sensorimotor Network connectivity at each stage of BP. However, no systematic review or meta-analysis has evaluated the neural mechanisms of acupuncture in PFNP using functional neuroimaging. Therefore, the results of our study will provide comprehensive evidence and help elucidate the neural mechanism of acupuncture for PFNP.

## Acknowledgments

We acknowledge the support and help of PROSPERO Review group and would like to appreciate the peer referees who provide review comments to improve the protocol.

## Author contributions

**Conceptualization:** Joo-Hee Kim.

**Investigation:** Dong Hyuk Lee, Joo-Hee Kim.

**Methodology:** Joo-Hee Kim.

**Writing – original draft:** Changwoo Seon, Dong Hyuk Lee, Joo-Hee Kim.

**Writing – review & editing:** Dong Hyuk Lee, Bo-In Kwon, Jun-Sang Yu, Sang Kyun Park, Yeonju Woo, Joo-Hee Kim.
